# Oncologic Brugada

**DOI:** 10.1016/j.jaccas.2024.102985

**Published:** 2025-01-02

**Authors:** Julia Bast, Mallette Asmuth, Nicole Prabhu, Lavanya Kondapalli, Raymundo A. Quintana

**Affiliations:** a Department of Medicine, University of Colorado Anschutz Medical Campus, Aurora, Colorado, USA; b Division of Cardiology, University of Colorado Anschutz Medical Campus, Aurora, Colorado, USA

## Abstract

Structural abnormalities within the right ventricular outflow tract (RVOT) can present similarly to Brugada syndrome. A 34-year-old woman with no medical history presented with polymorphic ventricular tachycardia/ventricular fibrillation cardiac arrest and initial electrocardiogram showed type I Brugada pattern. Cardiac magnetic resonance imaging revealed prominent tissue thickening at the RVOT with late gadolinium enhancement. Although imaging findings met criteria for arrhythmogenic right ventricular cardiomyopathy, thickening and infiltration in the anteroseptal RVOT is atypical, raising concern for an infiltrative process. Cardiac biopsy revealed primary cardiac diffuse large B-cell lymphoma.

## Graphical Abstract

**Figure:**
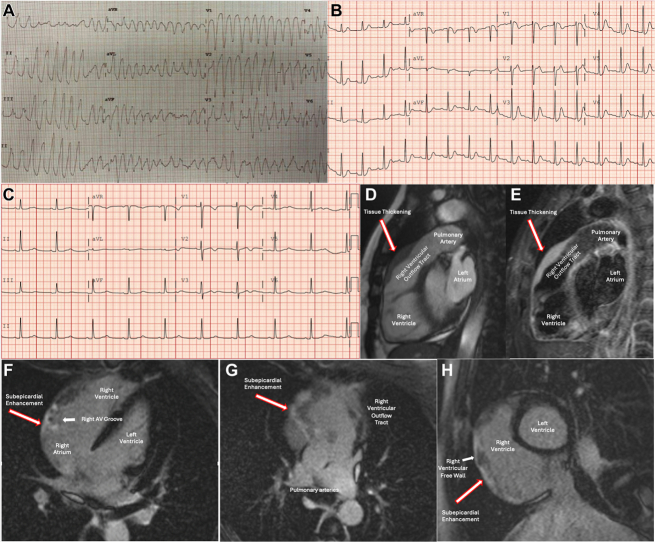

## Case Summary

**Figure 1:**
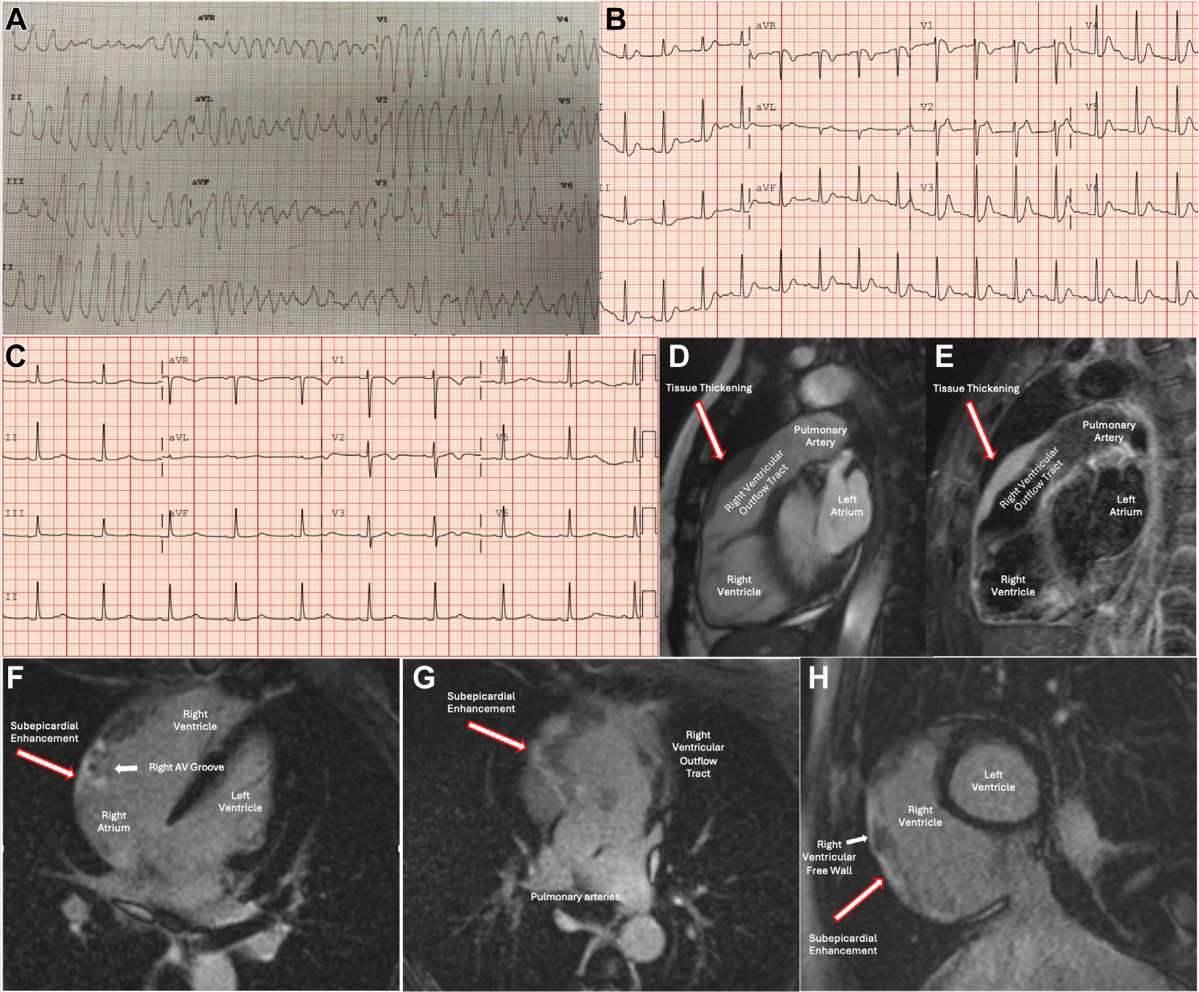
Cardiac Arrest Leading to an Unexpected Diagnosis (A) ECG obtained by EMS showing polymorphic VT. (B) Postresuscitation ECG showing normal sinus rhythm with coved ST-segment elevation in V_1_ followed by a negative T-wave. (C) Postchemotherapy ECG showing absence of Brugada pattern. (D) Isointense prominent tissue thickening of the anterior wall of RVOT on steady-state free precession. (E) Hyperintense tissue thickening on T2-weighted imaging. (F/G) Subepicardial LGE originating in the RV free wall, surrounding the right coronary artery, and extending into the RVOT. (H) Subepicardial LGE of the RV free wall. ECG = electrocardiogram; EMS = emergency medical services; LGE = late gadolinium enhancement; RV = right ventricle; RVOT = right ventricular outflow tract; VT = ventricular tachycardia.

A 34-year-old woman with no medical history collapsed while walking. Bystanders initiated cardiopulmonary resuscitation until emergency services arrived, and she received defibrillation twice for ventricular fibrillation with return of spontaneous circulation. While en route to the hospital, she developed sustained ventricular tachycardia (Figure 1A) and required additional defibrillation.

**Take-Home Messages**

• Brugada type 1 pattern on ECG can indicate electroanatomic and structural pathology localized to the RVOT. Therefore, cross-sectional imaging of the RVOT should be considered in patients presenting with this ECG pattern.
• Cardiac MRI enables detailed evaluation of malignant processes impacting the right AV groove.

On arrival at the emergency department, her vital signs were within normal limits and physical examination was unremarkable. Laboratory studies were notable for a potassium level of 4.5 mEq/L, magnesium of 1.8 mg/dL, bicarbonate of 14 mEq/L, lactate 6.5 mmol/L, and a negative urine drug screen. Postresuscitation electrocardiogram (ECG) (Figure 1B) showed sinus rhythm with coved ST-segment elevation in V_1_ followed by a negative T-wave, suggestive of type 1 Brugada pattern. Transthoracic echocardiogram was unremarkable with a left ventricular ejection fraction of 65%.

Cardiac magnetic resonance imaging (MRI) revealed the right ventricle (RV) was hypokinetic at the base, mildly dilated, and had a right ventricular ejection fraction of 42%. There was prominent tissue thickening involving the right atrioventricular (AV) groove and right ventricular outflow tract (RVOT) that was isointense on steady-state free precession (Figure 1D), hyperintense on T2-weighted imaging (Figure 1E), and had significant subepicardial late gadolinium enhancement (Figures 1F to 1H). There was no pericardial effusion, thoracic lymphadenopathy, or pulmonary metastasis. Imaging suggested an infiltrative process affecting the RV and RVOT such as a primary cardiac lymphoma or angiosarcoma. Coronary computed tomography ruled out coronary artery disease and showed the soft tissue in the right AV groove was encasing the right coronary artery, suggestive of lymphoma. Whole-body positron emission tomography revealed increased fluorodeoxyglucose uptake in the RV/RVOT and ruled out metastatic disease. The patient underwent sternotomy and open cardiac biopsy, which confirmed the diagnosis of a primary cardiac diffuse large B-cell lymphoma. A subcutaneous defibrillator was placed for secondary prevention. The patient underwent 6 cycles of rituximab, etoposide phosphate, prednisone, vincristine sulfate, cyclophosphamide, and doxorubicin hydrochloride and is currently in remission. Notably, her Brugada type 1 pattern on ECG resolved (Figure 1C).

The diagnosis of primary cardiac lymphoma is rare, accounting for only 1.3% of all cardiac tumors, with an overall incidence ranging from 0.0001% to 0.28%. Cardiac diffuse large B-cell lymphoma primarily affects elderly patients with only about 10% of patients being diagnosed before the age of 50 years. Brugada-like ECG pattern has been reported in cases of lymphoma with pericardial involvement, intracardiac metastatic tumors, and mediastinal tumors causing mechanical heart compression.^1^^,^^2^ This patient’s age, presentation of cardiac arrest, and primary cardiac lymphoma localized to the RVOT without pericardial involvement is a unique presentation. Electrical and micro-structural abnormalities within the RVOT are associated with Brugada pattern on ECG, which is the likely substrate for the patient’s presentation. Physicians should consider cross-sectional imaging of the RVOT in patients presenting with this ECG pattern.

Cardiac MRI is ideal for the evaluation of right-sided pathology and characterizing cardiac masses. Both cardiac lymphoma and cardiac angiosarcoma often involve the right AV groove. Angiosarcomas typically infiltrate the vessel wall and can occlude the right coronary artery, whereas lymphomas tend to grow around the right coronary artery, encasing the vessel.^3^

## Funding Support and Author Disclosures

The authors have reported that they have no relationships relevant to the contents of this paper to disclose.

## Appendix

Video 1 Oncologic Brugada A case of primary cardiac diffuse large B-cell lymphoma presenting as cardiac arrest and Brugada type 1 pattern on electrocardiogram
